# Antioxidant and Anti-tyrosinase Activities of Phenolic Extracts from Rape Bee Pollen and Inhibitory Melanogenesis by cAMP/MITF/TYR Pathway in B16 Mouse Melanoma Cells

**DOI:** 10.3389/fphar.2017.00104

**Published:** 2017-03-09

**Authors:** Liping Sun, Yan Guo, Yanxin Zhang, Yongliang Zhuang

**Affiliations:** Yunnan Institute of Food Safety, Kunming University of Science and TechnologyKunming, China

**Keywords:** rape bee pollen, phenolic composition, antioxidant properties, tyrosinase activity, glutathione synthesis, mRNA expression, cAMP

## Abstract

Rape bee pollen possesses many nutritional and therapeutic properties because of its abundant nutrimental and bioactive components. In this study, free (FPE) and bound (BPE) phenolic extracts of rape bee pollen were obtained, phenolic and flavonoid contents were determined, and composition of phenolic acids was analyzed. *In vitro* antioxidant and anti-tyrosinase (TYR) activities of FPE and BPE were compared, and inhibitory melanogenesis of FPE was further evaluated. Results showed FPE and BPE contain total phenolic contents of 11.76 and 0.81 mg gallic acid equivalents/g dry weight (DW) and total flavonoid contents of 19.24 and 3.65 mg rutin equivalents/g DW, respectively. Phenolic profiling showed FPE and BPE fractions contained 12 and 9 phenolic acids, respectively. FPE contained the highest rutin content of 774.87 μg/g. FPE and BPE showed the high antioxidant properties *in vitro* and high inhibitory activities for mushroom TYR. Higher activities of FPE than those of BPE can be attributed to difference in their phenolic compositions. Inhibitory melanogenesis activities of FPE against B16 were further evaluated. Results showed suppressed intracellular TYR activity, reduced melanin content, and promoted glutathione synthesis (*p* < 0.05) in FPE-treated cells. FPE reduced mRNA expression of TYR, TYR-related protein (TRP)-1 and TRP-2, and significantly suppressed cyclic adenosine monophosphate (cAMP) levels through down-regulation of melanocortin 1 receptor gene expression (*p* < 0.05). FPE reduced mRNA expression of microphthalmia-associated transcription factor (MITF), significantly inhibiting intracellular melanin synthesis (*p* < 0.05). Hence, FPE regulates melanogenesis of B16 cells involved in cAMP/MITF/TYR pathway. These results revealed that FPE can be used as pharmaceutical agents and cosmetics to protect cells from abnormal melanogenesis.

## Introduction

Bee pollen is a compound of floral pollen mixed with nectar and bee secretions, and contains carbohydrates, proteins, lipids, minerals, fibers, vitamins, and amino acids (Morais et al., 2011). Bee pollen is also rich in phytochemicals, such as phenolic compounds and particularly flavonoids (Mãrghitaş et al., 2009). Bee pollen presents many nutritional and therapeutic properties because of its abundant nutriments and bioactive components. Bee pollen is used as a health food as medicine, as officially recognized by the Pharmacopoeia Committee of the People’s Republic of China. Some studied showed that bee pollen improves the cardiovascular system, enhances body immunity, prevents prostate degeneration, maintains digestive system, and delays aging (Dong et al., 2015). During ancient times in China, bee pollen was used for skin whitening and beauty; however, only few studies reported this matter.

Phenolics recently received much attention for its wide range of different function, including antioxidant (Khatun et al., 2016; Xiao et al., 2016), anti-aging (Zhuang et al., 2017), antimicrobial (Alkan and Yemenicioǧlu, 2016), anti-diabetic (Xiao and Högger, 2015; Xiao et al., 2015), anti-hyperlipidemia (Sarma et al., 2016), hepatoprotective (Ma et al., 2015), and anti-inflammatory effects (Xiao, 2015). In addition, several researchers have reported the anti-tyrosinase and melanogenesis-inhibitory activities of various phenolic resources, including soybean (Shukla et al., 2016), *Ornithogalum narbonense* (Zengin et al., 2015), Mentha (Fatiha et al., 2015), and wild edible mushroom (Kaewnarin et al., 2016). Antioxidant activity of phenolics is mainly due to their redox properties hydrogen donors, and single-oxygen quenchers. Some studies reported that different phenolics present different activities, which are directly related with phenolic profiles. Bee pollen possesses wide range of phenolic compounds, such as rutin, quercetin, vanillic acid, and protocatechuic acid (Chu et al., 2007). However, bee pollen composition varies because of its botanical and geographic origins as well as other factors, such as soil type, weather conditions, and beekeeper activities (Feás et al., 2012).

Melanin is the main component responsible for skin disorders, such as agingspots, freckles, melisma, and malignant melanoma. Biosynthesis of melanin involves a series of complex oxidative and enzymatic reactions; tyrosinase (TYR) is one of the most important enzymes for melanin biosynthesis (Demirkiran et al., 2013). Antioxidant and TYR inhibitory activities are often selected as indicator of anti-melanogenesis. Many skin whitening agents pose anti-melanogenic effects through antioxidant activity and direct inhibitory effects on TYR activity or regulation of TYR expression. Previous studies showed that B16 mouse melanoma cell can be used to evaluate inhibitory melanogenic activities. Intracellular microphthalmia-associated transcription factor (MITF) is a key transcription regulator of genes responsible for melanin biosynthesis. MITF is also involved in regulation of melanocyte differentiation, pigmentation, proliferation, and survival. An important pathway involving MITF regulates production of melanin. Melanocortin 1 receptor (MC1R) increases levels of cyclic adenosine monophosphate (cAMP). Increasing cAMP induces transcription of MITF, which subsequently up-regulates expression of genes encoding for melanogenic enzymes, including TYR-related protein (TRP)-1 and TRP-2. This phenomenon thereby promotes melanin synthesis.

Rape plant is a vitally important economic crop in the world with high yields found in China. Rape bee pollen is used as nutritional food and traditional medicine with anti-inflammatory, antioxidant, anti-allergic, and cardioprotective activities (Lv et al., 2015). However, phenolic characteristics and anti-melanogenic functions of rape bee pollen in China are poorly understood. Therefore, free (FPE) and bound (BPE) phenolic extracts of rape bee pollen were prepared in this study. The present work determined phenolic contents and compositions as well as antioxidant and mushroom TYR inhibitory activities *in vitro*. Biological activity against B16 cell was evaluated. This study increases economic value of rape bee pollen.

## Materials and Methods

### Chemical and Reagents

The rape bee pollen was provided by the Sericulture and Apiculture Research Institute, Yunnan Academy of Agricultural Sciences (Mengzi, China). ABTS (2,2′-azino-bis-3-ethylbenzothiazoline-6-sulphonic acid), DPPH (2,2-diphenyl-1- picrylhydrazyl), TPTZ(2,4,6-tri-2-pyridinyl-1,3,5-triazine) and mushroom tyrosinase were obtained from Sigma–Aldrich Chemical Co. (St. Louis, MO, USA). Fetal bovine serum (FBS) was obtained from Sciencell Research Laboratories (San Diego, CA, USA). Dimethyl sulfoxide (DMSO), (4, 5-dimethylthiazol-2-yl)-2, 5-diphenyl tetrazolium bromide (MTT), trypsin-EDTA, Pen Strep solution (Penicillin 10000 units/mL and Streptomycin 10000 mg/mL) were purchased from Solarbio Co. (Beijing, China). Dulbecco’s Modified Eagle Medium (DMEM) + Gluta-MAXTM-I, Phosphate buffered saline (PBS) were purchased from Gibco Invitrogen (Thermo Fisher, USA). Acetonitrile and methanol used were HPLC grade, and other chemicals and solvents were of analytical grade.

### Extraction of Phenolics

Free phenolic extract and BPE of rape bee pollen were prepared. FPE of pollen was extracted per a previously described method with some modifications (Nardini and Ghiselli, 2004). Lipids from samples (2 g) were removed with hexanes, and subsequently extracted with 100 mL of 70% methanol by ultrasound for 60 min. Extract was homogenized using a TGL-20B homogenizer at 4,000 rpm for 15 min. Supernatants were diluted twice with water and acidified to pH 2 with 2 M HCl. Extract was then extracted four times with ethyl acetate. Ethyl acetate supernatants were pooled and 10 g Na_2_SO_4_ was added afterward, Sample was then filtered and evaporated at 45°C to reach a dry state. FPE were reconstituted with methanol for analysis.

Pollen extraction of BPE was conducted based on a previous study (Wang et al., 2011). Residue from free phenolics extraction was hydrolyzed with 100 mL of 2 M NaOH at room temperature for 1 h with continuous shaking under nitrogen gas. Extract was homogenized using a TGL-20B homogenizer at 4,000 rpm for 15 min. Supernatants were diluted twice with water and acidified to pH 2 with 2 M HCl. Extract was then extracted four times with ethyl acetate. Ethyl acetate supernatants were pooled and 10 g Na_2_SO_4_ was added afterward. Sample was filtered and evaporated at 45°C to reach a dry state. BPE was reconstituted with methanol for analysis.

### Determination of Total Phenolic Contents

Total phenolic contents were analyzed by the Folin-Ciocalteu (FC) colorimetric method (Moreira et al., 2008) with slight modifications. Briefly, 0.5 mL extract was allowed to react with 2.5 mL of 10% FC reagent for 5 min, and 2 mL of 7.5% aqueous sodium carbonate solution was subsequently added. Reaction in mixture lasted for 60 min at room temperature in the dark. Absorbance was detected at 765 nm using a TU-1901 spectrometer (Persee Inc., Beijing, China) with gallic acid as standard. Total phenolic content was expressed as mg gallic acid equivalents (GAE) per 1 g dry weight (DW) of samples.

### Determination of Total Flavonoids Content

Total flavonoid contents were determined by NaNO_2_-Al(NO_3_)_3_ method. Extract was mixed with 0.3 mL 5% NaNO_2_ solution for 6 min followed by addition of 0.3 mL 10% Al(NO_3_)_3_ solution for 6 min. 4 mL of 1 M NaOH solution was then added to the reaction mixture. Ethanol was incorporated to obtain 10 mL total volume. Absorbance was determined at 510 nm using rutin as standard. Total flavonoid content was expressed as mg rutin equivalents (RE) per 1 g DW of sample.

### Determination of Phenolic Composition

High performance liquid chromatography (Agilent 1000, Agilent Technologies, Santa Clara, CA, USA) of phenolic compounds was performed on a reverse-phase Zorbax SB-C18 column (4.6 mm × 250 mm, 5-micron), using a gradient program with two solvent systems (A, 0.1% acetic acid in water; B, 0.1% acetic acid in acetonitrile). The initial condition was 8% B; it was changed to 10% B (0–2 min); to 30% B (2–27 min), to 90% B (27–50 min), to 100% B (50–51 min), to 100% B (51–55 min), to 8% B (55–58 min). Injection volume was 20 μL. Flow rate was 1 mL/min and signals were detected at 280 nm (Sun et al., 2014). Calibration curves were plotted using five different concentrations of 12 phenolic compound standards.

### Antioxidant Activities *In vitro*

#### DPPH Radical Scavenging Activity

Scavenging DPPH radical activity was determined by a previously described method (Wang et al., 2012). Briefly, 0.4 mL of samples were allowed to react with 2 mL of 0.1 mM methanolic solution of DPPH radical. The mixture was then evenly combined and allowed to stand at room temperature in the dark for 30 min. Absorbance was measured at 517 nm against methanol blank. Vitamin C (Vc) was used as a positive control. DPPH radical scavenging ability was expressed as percentage inhibition of DPPH radical. Inhibitory percentage was calculated using the equation below:

$$\text{Scavenging ability}(\%)=(1-A_{\text{sample}}/A_{\text{blank}})\times 100\,$$

IC_50_ value was defined as effective concentration required for scavenge 50% radical.

#### ABTS_●+-_Scavenging Activity Assay

Stock 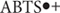 solution was prepared from 7 mM ABTS and 40 mM of potassium persulfate in distilled water (Re et al., 1999). When used in 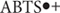 working solution, stock 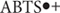 solution was diluted with ethanol to achieve an absorbance of 0.75 at 734 nm. Briefly, 4 mL of 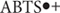 working solution and 0.5 mL of sample solution of different concentrations were mixed and incubated at 30°C in water bath for 6 min. Absorbance at 734 nm was recorded. Trolox was used as positive control. 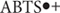-scavenging activity was calculated by Formula (1). IC_50_ value was defined as an effective concentration for scavenge 50% radical.

### Reducing Power Assay by Ferric Reducing Antioxidant Power (FRAP) Method

Freshly prepared FRAP reagent contained 100 mL of acetate buffer (300 mM, pH 3.6), 10 mL of TPTZ solution (10 mM), and 10 mL of ferric chloride solution (20 mM). Briefly, 4.5 mL of FRAP reagent and 0.15 mL of sample solution of different concentrations were mixed and incubated at 37°C in water bath for 6 min. Absorbance at 593 nm was recorded. Increase in A593 represents reducing power (Benzie and Strain, 1996). Vc was used as positive control. EC_50_ was defined as effective mixture concentration that produces 0.500 absorbance units.

### Determination of Inhibitory Activity for Mushroom TYR

Distilled water was added to different concentrations of samples to reach 1 mL volume. After 5 min of incubation at room temperature, 3 mL of L-tyrosine was mixed and incubated for 15 min at 37°C. Afterward, 0.1 mL of mushroom TYR solution was added and incubated for 25 min at 37°C. Absorbance was measured at 470 nm against blank without TYR. Percentage inhibition of enzyme activity and IC_50_ values were calculated according to the following equation:

$$\text{Inhibition}(\%)=(A_{0}-A_{1}+A_{2})/A_{0}\times 100\,\,$$

where *A*_0_, *A*_1_, and *A*_2_ are absorbance values of control, samples, and blank, respectively.

### Cell Culture and Determination of Intracellular activity

#### Cell Culture

B16 mouse melanoma cells were purchased from the Kunming Institute of Zoology of the Chinese Academy of Sciences (Kunming, China). The B16 melanoma cells were cultured in high glucose-DMEM, and then supplemented with 10% FBS, 100 mg/mL streptomycin and 100 U/mL penicillin. Cells were maintained in a humidified incubator with 5% CO_2_ at 37°C, and were sub-cultured every 2 days to maintain logarithmic growth.

#### Cell Viability

B16 melanoma cell viability was assessed using a previously described method with slight modification (Souza et al., 2012). Cells (1.0 × 10^4^ cells, 200/μL⋅well) were seeded into 96-well microplates. After 24 h of incubation, different concentrations (0, 20, 60, and 100 μg/mL) of samples, along with 200 nM α-melanocyte stimulating hormone (α-MSH), were added to each well of plates. After incubating plates for additional 24 h and 48 h, attached cells were incubated with MTT (0.5 mg/mL, 4 h) and subsequently solubilized in DMSO. Absorbance at 570 nm was then measured using a micro-plate reader (SpectraMax M5 Multi-Mode Microplate Reader, Molecular Devices, Sunnyvale, CA, USA) to calculate percentage of cell viability.

### Measurement of Melanin Contents

Melanin content was measured referring to a previously described method with slight modification (Tsuboi and Kondoh, 1998). B16 cells (2 × 10^5^ cell/well) were grown in six-well plates. With different concentration samples and 200 nM α-MSH added for 48 h, cells were dissolved in 1 mL of 1 M NaOH (containing 10% DMSO) by ultrasound for 30 min, incubated (90°C) in water bath for 2 h, and centrifuged for 15 min at 3,000 rpm. Optical densities (OD) of supernatants were measured at 450 nm using an ELISA reader. Intracellular protein content was measured by BCA method. Relative melanin content was calculated using the following equation:

$$\text{Melanin relative contents}(\%)={\text{OD}}_{1}/{\text{OD}}_{0}\times 100\,\,$$

The results were fixing by protein content. OD_0_ and OD_1_ were ODs of control and samples, respectively.

### TYR Activity Assay

Activity assay for intracellular TYR was performed using a previously described method (Tomita, 1992). B16 melanoma cells (2 × 10^5^ cells/well) were incubated in six-well plates with different sample concentrations and 200 nM α-MSH. Cells were washed twice with PBS (0.1 M, pH 6.8), freeze-thaw lysed in 300 μL of 0.1 M PBS containing 5% Triton X-100 and ultrasound-lysed in a ice-water bath. Briefly, 200 μL of supernatants were incubated at 37°C for 10 min and then mixed with 50 μL of 0.1% L-DOPA in PBS (0.1 M, pH 6.8). Spectrophotometric analysis was performed at 475 nm for 0 min and 30 min; content of protein was measured by BCA method. TYR-related activity was calculated using the following equation:

$$\text{TYR relative activity}(\%)=(A_{30}^{\prime }-A_{0}^{\prime })/(A_{30}-A_{0})\times 100$$

Results were fixed by protein content. *A*′_0_, *A*′_30_ represent absorbance values of samples at 0 and 30 min, respectively, where as *A*_0_ and *A*_30_ are absorbance values of control at 0 and 30 min, respectively.

### Ratio of Glutathione and Glutathione Disulfide (GSH/GSSG) Assay

Glutathione and glutathione disulfide ratio was determined using the corresponding kits (NanJing JianCheng Bio Institute, Nanjing, China). GSH content was determined following formation of yellow-colored 5-thio-2-nitrobenzoic acid (TNB), which is produced by reaction between GSH and 5, 5′-dithiobis-2-nitrobenzoic acid (DTNB). GSSG was measured according to DTNB-glutathione reductase recycling assay (Hou et al., 2011). Total protein content was measured according to BCA method, and all results were fixed by protein content.

### Determination of Intracellular cAMP Levels

B16 melanoma cells were treated similar to the TYR activity assay. Intracellular cAMP levels were measured using an I 125 kit (Shanghai University of Chinese Traditional Medicine, Shanghai, China) following manufacturer’s instructions.

### Measurement of mRNA Expression by Quantitative Reverse Transcription-Polymerase Chain Reaction (qRT-PCR)

B16 cells (8 × 10^4^ cells/well) were cultured in 2 mL of six-well plate for 24 h. After treatment with different concentrations of FPE and α-MSH (200 nM) for 48 h, cells were washed with cold PBS. Total RNA was extracted by Trizol. RT was conducted by PrimeScript^TM^ RT reagent Kit with gDNA Eraser (Perfect Real Time) (TaKaRa Biotechnology, Dalian, China) following manufacturer’s instructions. cDNA was amplified on StepOnePlus Real-Time PCR System (Applied Biosystems, Foster City, CA, USA) using SYBR^®^ Premix Ex Taq^TM^ II (Tli RNaseH Plus) (TaKaRa Biotechnology, Dalian, China) and corresponding probes (Shenggong, Shanghai, China). Real-time qPCR analysis was conducted under the following temperatures and times: initial denaturation at 95°C for 30 s and 40 cycles of denaturation at 95°C for 5 s and annealing at 60°C for 30 s. Relative expression level of each melanogenic protein mRNA was normalized to β-actin mRNA. Primers refer to the following previously reported primer sequences as follows: For β-actin, 5′-ACTATTGGCAACGAGCGGTT-3′(forward) and 5′-ATGGATGCCACAGGATT-CCA-3′(reverse); for TYR, 5′-GTCGT ACCCTGAAAATCCTAACT-3′(forward) and 5′-CATCG-CATAAAACCTGATGGC3′(reverse); for TRP-1, 5′-CTTTCTCCCTTCCTTACTGG-3′ (forward) and 5′-TCGTACTCTTCCAAGGATTCA-3′(reverse); for TRP-2, 5′-TTATATCCTTCGAAA-CCAGGA-3′ (forward) and GGGAAT-GGATATTCCGTCTTA-3′ (reverse); for MITF, 5′-GTATGAACACGCACTCTCGA-3′(forward) and 5′-GTAACGTATTTGCCATTTGC-3′ (reverse) (Kwak et al., 2011); for MC1R, 5′-TGACCTGATGGTAAGTGTCAGC-3′ (forward) 5′-ATGAGCACGTCAATGAGGTT-3′ (reverse) (Chang et al., 2015).

### Statistical Analyses

Data were expressed as means ± standard deviation, and were tested using SPSS (Version 20.0, SPSS Inc., Chicago, IL, USA). Differences at *p* < 0.05 were considered significant.

## Results and Discussion

### Total Phenolic and Flavonoid Contents

In this study, FPE and BPE were obtained. As shown in **Table 1**, FPE and BPE of rape bee pollen were 11.76 and 0.81 mg GAE/g DW, respectively. FPE comprised a large percentage (93.5%) of total phenolic content. Our result was higher than that of Žilić et al. (2014), who reported that in different genotypes of maize pollen, total phenolics were within 7.78–9.93 mg GAE/g DW. Total flavonoid content was 19.24 mg RE/g DW, which was higher than that of maize pollen (Žilić et al., 2014). Free flavonoid content also accounted for large percentage (84.1%) of total flavonoid in rape bee pollen.

**Table 1:** Determination of total phenolic and flavonoid content of rape bee pollen.

	Total phenolic content (mg GAE/g DW)	Total flavonoid content (mg RE/g DW)
FPE	11.76 ± 0.04^b^	19.24 ± 0.06^b^
BPE	0.81 ± 0.01^c^	3.65 ± 0.03^c^
Total	12.57 ± 0.05^a^	22.89 ± 0.09^a^


*Different letters in the same column were significantly different (*p* < 0.05).*

### Phenolic Compositions

As shown in **Table 2**, 12 and 9 phenolic acids were found in FPE and BPE, respectively. FPE contained high quantity of rutin (774.87 μg/g). Similar quantities of rutin were measured in bee pollen samples from Baltic region (Kaškonienë et al., 2015). Wang et al. (2012) reported that aqueous extracts of green asparagus exhibit significant antioxidant and anti-tyrosinase activity; such extracts are rich in rutin. In rape bee pollen, quercetin content of FPE was 196.38 μg/g, which was close to that of honeybee pollen collected in the *Baltic* region (Kaškonienë et al., 2015). Quercetin is a flavonoid that exhibits high antioxidant and anti- melanogenesis activities. High quantity of benzoic acid was detected in free extract (314.16 μg/g), and kaempferol content was 9.26 μg/g; these values are higher than those reported of Fanali et al. (2013). Resveratrol content in FPE was 242.88 μg/g. Our result was higher than that reported by Cheng et al. (2013), who reported Shanxi (China) bee pollen contained 177.15 μg/g resveratrol. Resveratrol possesses a number of beneficial effects, including anticancer, antiatherogenic, antioxidative, anti-inflammatory, anti-microbial and estrogenic activities. Cinnamic acid was found in BPE and was particularly high in FPE (102.65 μg/g); this amount is higher than that for some previously described pollen extracts (Kaškonienë et al., 2015). *P*-coumaric acid content was higher than the pollen covered (Ulusoy and Kolayli, 2014). In sum, rape bee pollen presents a wide range of variety because of its phenolic acids components, which differ from those of other pollens. Floral origin, geographical collection area, and bee species are elements responsible for imparities in qualitative and quantitative composition of bee pollen phenolic compounds.

**Table 2:** Phenolic composition of different extracts of rape bee pollen.

Phenolic compounds	FPE (μg/g)	BPE (μg/g)
Rutin	774.87 ± 8.77	6.45 ± 0.40
*p*-hydroxybenzoic acid	84.28 ± 5.29	11.08 ± 0.13
Benzoic acid	314.16 ± 11.87	3.46 ± 0.14
Resveratrol	242.88 ± 6.32	4.39 ± 0.13
Quercetin	196.38 ± 3.14	nd
Cinnamic acid	102.65 ± 3.79	2.30 ± 0.17
Vanillin	58.41 ± 1.22	nd
Kaempferol	9.26 ± 6.21	0.17 ± 0.18
Protocatechuic acid	119.38 ± 4.82	nd
*p*-coumaric acid	32.63 ± 2.19	11.22 ± 0.10
Gallic acid	tr	tr
Catechin	tr	tr


*nd, not detected; tr, trace.*

### Antioxidant Activities *In vitro*

Various methods were used to analyze antioxidant capacity because antioxidant agents with different compositions and contents present different mechanism for their antioxidant capacities. In the present study, we selected three models in evaluating antioxidant activities of phenolic extract of rape bee pollen *in vitro*, such activities include scavenging radical activity of DPPH and ABTS assays and ferric-ion-reducing capacity.

2,2-diphenyl-1- picrylhydrazyl assay is widely used in evaluating ability of antioxidant to scavenge free radicals (Shukla et al., 2016). 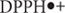-stable purple radicals react with antioxidants, generating stable diamagnetic molecule. Solutions lose their color per the number of electrons used up (Fatiha et al., 2015). As shown in **Table 3**, FPE showed high DPPH scavenging ability and IC_50_ value of 1.27 μg/mL. FPE demonstrated better activity than BPE and Vc, suggesting that FPE are good DPPH scavengers.

**Table 3:** Antioxidant capacity and mushroom tyrosinase inhibitory activity of rape bee pollen.

	DPPH IC_50_ (μg/mL)	ABTS IC_50_ (μg/mL)	FRAP EC_50_ (μg/mL)
FPE	1.27 ± 0.00^c^	3.19 ± 0.06^c^	0.12 ± 0.00^c^
BPE	3.21 ± 0.04^a^	3.85 ± 0.02^b^	2.98 ± 0.02^a^
Positive control	3.05 ± 0.61^b^	45.88 ± 0.25^a^	1.05 ± 0.5^b^
	(Vc)	(Trolox)	(Vc)


*Different letters in the same column were significantly different (*p* < 0.05).*

In ABTS assay, blue/green 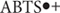 chromophore is produced by reaction of ABTS and potassium persulfate. Antioxidants reduce radical of 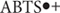 back to ABTS (Guedes et al., 2013). Phenolic extract of rape bee pollen showed high ABTS radical-scavenging activity. FPE and BPE yielded IC_50_ values of 3.19 and 3.85 μg/mL, respectively (**Table 3**). FPE exhibited higher antioxidant capacity and significantly better than Trolox (45.88 μg/mL).

Ferric reducing antioxidant power assay is based on ability of antioxidant to reduce Fe^3+^ to Fe^2+^ in the presence of TPTZ (Zengin et al., 2015), forming an intense blue Fe^2+^-TPTZ complex with maximum absorption at 593 nm. Reduced absorbance is proportional to antioxidant content. **Table 3** shows results for ferric-ion-reducing activities of rape bee pollen after FRAP assay. FRAP capacity followed the sequence, FPE > Vc > BPE. FPE demonstrated significantly higher activity than BPE (*p* < 0.05) and EC_50_ value of 0.12 μg/mL. FPE of rape bee pollen presents better reducing power.

### Mushroom TYR Inhibitory Activity

**FIGURE 1:**
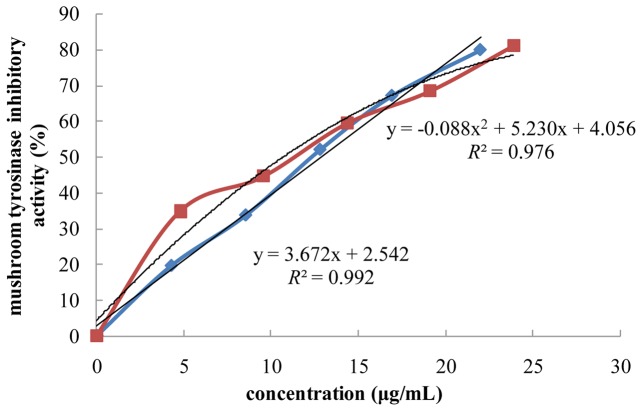
**Effect of phenolic extracts of rape bee pollen on mushroom tyrosinase inhibitory activity. 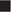: FPE, 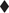: BPE**.

Tyrosinase inhibitors are clinically used for treatment of some dermatological disorders associated with melanin hyperpigmentation and for skin whitening in cosmetic. As shown in **Figure 1**, FPE showed highly potent inhibition against mushroom TYR activity. The following equation expresses formula for anti-tyrosinase activities (y) of FPE and its concentrations (x): *y* = -0.088x2+5.230x+4.056; IC_50_ value was calculated as 10.72 μg/mL. Anti-tyrosinase activities and concentrations of BPE exhibited linear correlation; *R*^2^ was 0.992, and calculated IC_50_ value was 12.92 μg/mL (**Figure 1**). FPE showed higher activity than BPE. Inhibitory activity against tyrosinase was also observed for some phenolic compounds, such as quercetin, benzoic acid and their derivatives. Therefore, inhibitory tyrosinase activity of FPE and BPE is related to their phenolic profiles.

Considering *in vitro* antioxidant and anti-tyrosinase activities of FPE and BPE, we further evaluated the effect of FPE on anti-melanogenesis in B16 mouse melanoma cells.

### Cell Viability

**FIGURE 2:**
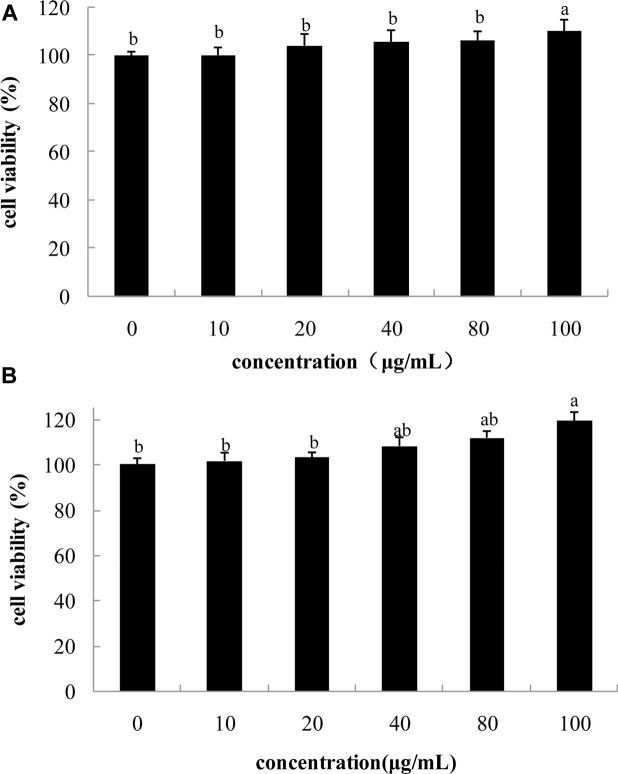
**Effect of phenolic extracts of rape bee pollen on cell viability of B16 cells,** **(A)** 24 h, **(B)** 48 h. Different letters were significantly different (*p* < 0.05).

Any potential cytotoxic effect of rape bee pollen extracts should be determined before further testing (Chang et al., 2015). Effect of FPE on viability of B16 cells was measured. As shown in **Figure 2**, FPE did not reduce growth of B16 melanoma cells at concentration of 0–100 μg/mL. This result revealed that rape bee pollen extracts are non-cytotoxic to B16 cells.

### Intracellular Melanin Synthesis and TYR Activity in Melanocytes

Intracellular melanin content directly reflects anti-melanogenic activity. **Table 4** shows different concentrations of FPE inhibiting increase in melanin content in B16 cells. Phenolic extracts of rape pollen potently suppressed melanin production in B16 cells. FPE affects melanin content in a dose-dependent manner. Inhibitory melanin production activity increased with increasing concentrations of FPE. Intracellular melanin contents significantly decreased by 47.51% at concentration of 100 μg/mL.

**Table 4:** Effects of free rape bee pollen extract on intracellular melanin content and tyrosinase inhibitory activity.

Concentration (μg/mL)	Melanin relative contents (%)	Tyrosinase relative activity (%)
control	100 ± 1.21*^a^*	100 ± 3.25*^a^*
20	84.23 ± 2.16*^b^*	95.39 ± 4.84*^a^*
60	67.37 ± 2.23*^c^*	72.49 ± 4.17*^b^*
100	52.49 ± 3.06*^d^*	59.14 ± 4.02*^c^*


*Different letters in the same column were significantly different (*p* < 0.05) from each other.*

Tyrosinase is a vital enzyme for melanin synthesis, and observing its intracellular activity is constantly the first step in studying melanin synthesis (Hu et al., 2015). As shown in **Table 4**. FPE effectively inhibited intracellular TYR activity of B16 cells in dose-dependent manner; this inhibition is similar to change in melanin content. At concentration of 100 μg/mL, FPE significantly reduced TYR activity in B16 cells by 40.86%. TYR inhibitory activities of rape bee pollen extract are associated with its phenolic acids and antioxidant activities. This finding is similar to that of a previous study (Shukla et al., 2016), which reported that TYR inhibitory activity is related to hydroxyl group of phenolic compounds. This group forms hydrogen bonds with enzyme active sites to promote steric hindrance, conformational changes, and directly suppress enzymatic activity.

### GSH/GSSG Value

**FIGURE 3:**
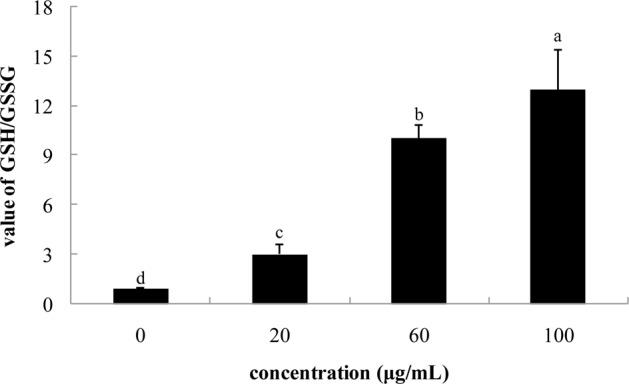
**Effect of phenolic extracts of rape bee pollen on the value of GSH/GSSG of B16 cells.** Different letters were significantly different (*p* < 0.05).

Melanin synthesis is involved in production of several reactive oxygen species, including ●O^2-^, ●OH, and NO●. Therefore, intracellular reducing power plays a crucial role in the regulation of melanogenesis (Zhuang et al., 2009). As an important intracellular indicator, the GSH/GSSG ratio is involved in the intracellular regulation of melanin synthesis. **Figure 3** shows effects of pollen phenolic extract on intracellular GSH/GSSG ratio in B16 cells observed in this study. Phenolic extracts significantly enhanced intracellular GSH/GSSG ratio, and value of GSH/GSSG dose-dependently increased with concentration of FPE. Specifically, value of GSH/GSSG in B16 treated with FPE (100 μg/mL) was 13.55 times that of control group. TYR activity in B16 cell requires oxidative pressure. Phenolics effectively increase reducing power of B16 cell via their antioxidant activity. Therefore, phenolics of FPE indirectly inhibit intracellular activity of TYR to reduce melanin synthesis.

### Intracellular mRNA Expression of TYR, TRP-1 and TRP-2

**FIGURE 4:**
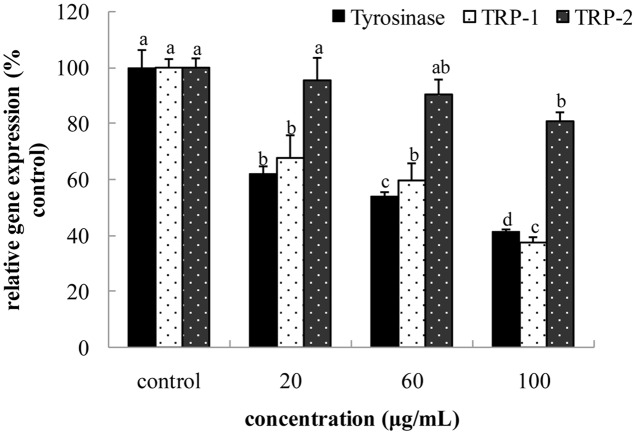
**Effect of free phenolic extract (FPE) of rape bee pollen on tyrosinase (TYR), TRP-1 and TRP-2 of B16 cells.** Different letters were significantly different (*p* < 0.05) in same bar graph.

Tyrosinase, TRP-1 and TRP-2 are key enzymes involved in melanin biosynthesis. TYR catalyzes two distinct reactions, including conversion of tyrosine to L-DOPA and DOPA to dopaquinone. Dopaquinone is spontaneously converted to dopachrome. TRP-2 catalyzes conversion of dopachrome to 5, 6-dihydroxyindole-2-carboxylic acid (DHICA), whereas TRP-1 induces oxidation of DHICA to indole-5, 6-quinone-2-carboxylic acid. As shown in **Figure 4**, after treatment with FPE at concentrations of 20, 60, and 100 μg/mL, TYR expression in B16 melanoma cells significantly reduced by 37.18, 46.05, and 58.47%, respectively, in a dose-dependent manner. Expression levels of TRP-1 were inhibited by 32.49, 40.40, and 61.63%, respectively. Compared with control, effect of 20 μg/mL FPE was not significantly different on TRP-2 (*p* > 0.05), and that of 100 μg/mL FPE was significantly lower than that of control (*p* < 0.05).

### Intracellular Content of cAMP and the mRNA Expression of MC1R and MIFT

**FIGURE 5:**
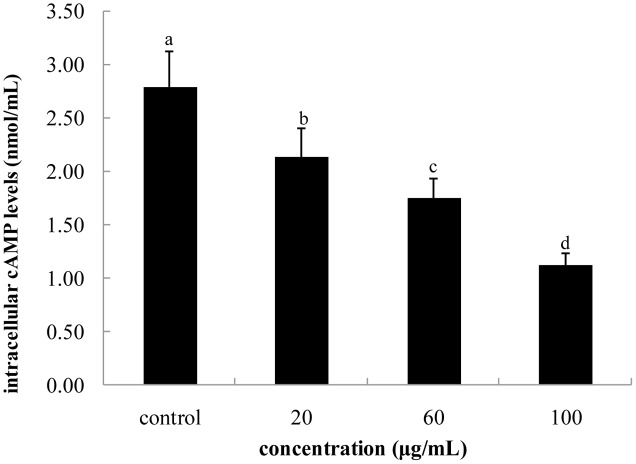
**Effect of FPE of rape bee pollen on intracellular cAMP levels of B16 cells.** Different letters were significantly different (*p* < 0.05).

**FIGURE 6:**
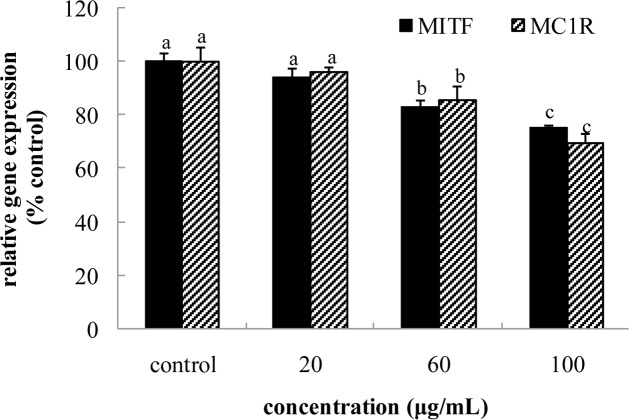
**Effect of FPE of rape bee pollen on microphthalmia-associated transcription factor (MITF) and MC1R mRNA expression of B16 cells.** Different letters were significantly different (*p* < 0.05) in same bar graph.

Microphthalmia-associated transcription factor regulates expression of MC1R and α-MSH binds to its specific MC1R, which enhances MITF expression. Expression of MC1R increases levels of cAMP, and cAMP pathway plays a key role in regulation of melanogenesis. cAMP-elevating agents enhance expression of TYR mRNA, implying requirement of cAMP for optimal melanogenic activity. As shown in **Figures 5**, **6**, both intracellular cAMP levels and MC1R expression in FPE-treated B16 cells were down-regulated in a dose-dependent manner. Compared with control, cAMP levels and MC1R expression decreased by 59.71 and 30.62%, respectively, at FPE concentration of 100 μg/mL. Increase in cAMP level induced MITF transcription. MITF is a pivotal regulator involved in expression of melanogenic proteins. As shown in **Figure 6**, expression level of MITF was reduced by FPE in a dose-dependent manner. Compared with control, expressions of MITF reduced by 16.89 and 24.92% at 60 and 100 μg/mL FPE treatment, respectively. FPE induced significant suppressions on mRNA expression levels of TYR via MITF down-regulation, which was caused by reduced intracellular cAMP levels. These results revealed that melanogenesis inhibitory mechanism of FPE is involved in cAMP/MITF/TYR pathway.

## Conclusion

In this study, we determined total phenolic and flavonoid content and analyzed phenolic acid compositions from FPE and BPE of rape bee pollen from Yunnan, China. Results showed that phenolics of rape bee pollen are different from previously reported kinds of pollen from various areas. Phenolic extracts of rape bee pollen showed strong antioxidant properties and anti-TYR activity *in vitro*; activities of free form were higher than those of bound form. FPE reduced melanin content, inhibited activity of TYR, and increased GSG/GSSG in B16 melanoma cells. FPE down-regulated expressions of TYR, TRP-1, and TRP-2 through diminution of expression or activation of MITF. Exposure to FPE caused reduction in cAMP levels through down-regulation of MC1R gene expression, which exerts a negative regulatory role in melanogenesis. Therefore, inhibitory melanogenesis activity of FPE is regulated by cAMP/MITF/TYR pathway. FPE of rape bee pollen can be used as a source of natural anti-melanogenesis composition.

## Author Contributions

LS and YZhu were involved in the project design, carried out most of the experiments, and drafted the manuscript. YZha participated to extract the phenolic from rape bee pollen and evaluate *in vitro* antioxidant activity. YG and YZha contributed to the cell experiment and data analysis. All authors read and approved the manuscript finally.

## Conflict of Interest Statement

The authors declare that the research was conducted in the absence of any commercial or financial relationships that could be construed as a potential conflict of interest.
